# Insulin expression in β cells is reduced within islets before islet loss in diabetic cats

**DOI:** 10.1111/jsap.13541

**Published:** 2022-08-19

**Authors:** V. Bergomi, S. Beck, M. Dobromylskyj, L. J. Davison, J. W. Wills, K. Hughes

**Affiliations:** ^1^ Department of Veterinary Medicine University of Cambridge Cambridge CB3 0ES UK; ^2^ VPG Histology Horner Court Bristol BS7 0BJ UK; ^3^ Finn Pathologists Harleston IP20 9EB UK; ^4^ Department of Clinical Sciences and Services Royal Veterinary College Hatfield UK; ^5^ Wellcome Centre for Human Genetics University of Oxford Oxford UK; ^6^ Mercer & Hughes Veterinary Surgeons Saffron Walden CB11 3JB UK; ^7^ Independent Anatomic Pathology Ltd Bath UK

## Abstract

**Objectives:**

Diabetes mellitus is a common condition that requires intensive treatment and markedly impacts the welfare of affected cats. The aim of this study was to identify diabetes mellitus‐associated perturbations in the feline pancreatic islet microenvironment. The utility of “clear, unobstructed brain/body imaging cocktails and computational analysis” (CUBIC) for three‐dimensional pancreatic analysis was investigated.

**Methods:**

Formalin‐fixed paraffin‐embedded tissues from cats with diabetes mellitus, or control cats without pancreatic pathology, were retrospectively identified. Immunohistochemistry for synaptophysin and ionised calcium binding adaptor molecule 1, and immunofluorescence for insulin and synaptophysin, were used to assess changes in islets. An image analysis pipeline was developed to analyse images acquired from two‐dimensional immunofluorescence. CUBIC was used to optically clear selected pancreas samples before immunofluorescence and deep three‐dimensional confocal microscopy.

**Results:**

Diabetic cats have a significant reduction in synaptophysin‐positive islet area. Whilst islets from diabetic patients have similar numbers of β cells to islets from control cats, significantly lower intensity of insulin expression can be observed in the former. CUBIC facilitates clear visualisation of pancreatic islets in three dimensions.

**Clinical Significance:**

The data presented support the theory that there is a decrease in function of β cells before their destruction, suggesting a potentially significant step in the pathogenesis of feline diabetes mellitus. In parallel, we demonstrate CUBIC as a valuable new tool to visualise the shape of feline pancreatic islets and to interrogate pathology occurring in the islets of diabetic pets.

## INTRODUCTION

Diabetes mellitus (DM) is a commonly encountered endocrinopathy in cats, with 1 in 230 cats affected in the United Kingdom (O'Neill *et al*. 2016). DM has a significant welfare impact on affected animals as it requires intensive and often lifelong treatment. There are frequently ensuing complications, such as urinary tract infections, peripheral neuropathy and diabetic ketoacidosis (Kramek *et al*. 1984, Mizisin *et al*. 2002, Bailiff *et al*. 2006, Mayer‐Roenne *et al*. 2007, Olin & Bartges 2015). Feline DM is often compared with human type 2 diabetes mellitus (T2DM) as both are characterised by insulin resistance and impaired β cell function, due to a combination of genetic and environmental factors (Dixon *et al*. 2002, Henson & O'Brien 2006, Nelson & Reusch 2014). Whilst obesity is a key risk factor for feline diabetes, the presence of pancreatitis or hypersomatotropism can also contribute to the development of the diabetic phenotype. In a state of insulin resistance, transport of glucose into cells, for use as a source of energy, is impaired, exacerbating hyperglycaemia. A state of persistent hyperglycaemia can lead to various degrees of glucotoxicity, which is detrimental to the function of islet cells, and may ultimately lead to their destruction. Glucotoxicity can be transient and therefore reversible, but where islets are susceptible and hyperglycaemia is severe or persistent, permanent islet damage may occur (O'Brien 2002, Henson & O'Brien 2006, Rotlewicz *et al*. 2010, Osto *et al*. 2013, Samaha *et al*. 2019).

Understanding the pathogenesis of feline DM is a key step in identification of new therapeutic targets and is thus essential to improve prognosis for diabetic patients. The clinical observation that diabetic cats may enter clinical remission once insulin therapy is initiated supports a pancreatic beta cell dysfunction rather than beta cell destruction model of disease pathogenesis, at least in the early stages of disease. As the pathology of DM primarily affects pancreatic islets, it logically follows that the islet microenvironment should be closely evaluated, both in healthy and diabetic states (Bruskiewicz *et al*. 1997, Cooper *et al*. 2015). A recent histopathological study of feline endocrine pancreas highlighted a significant loss of β‐cell mass in the absence of notable islet inflammation in cats with DM (Zini *et al*. 2016), consistent with a T2DM phenotype. Islet amyloidosis has also been observed with varying frequency in diabetic cats (22% to 100%) (O'Brien *et al*. 1993, Henson & O'Brien 2006, Osto *et al*. 2013, Zini *et al*. 2016) although a proportion of older non‐diabetic cats can also present with islet amyloidosis, making its role in the pathogenesis of DM unclear (Herndon *et al*. 2014, Zini *et al*. 2016). Thus, whilst some key changes in the islet microenvironment of diabetic cats have been delineated, many questions remain regarding how and why a healthy islet undergoes transformations that ultimately culminate in loss of function.

In order to further interrogate the islet microenvironment to better understand the pathogenesis of DM in cats, we have employed two‐dimensional (2D) immunohistochemistry (IHC) and immunofluorescence (IF) to compare the islet microenvironment of feline DM patients with that of cats with no clinical history indicating DM. We have also capitalised upon the emergence of a tissue clearing method termed “clear, unobstructed, brain imaging cocktail and computational analysis” (CUBIC). This methodology renders tissues transparent which, when combined with IF to label structures of interest, facilitates deep 3D visualisation of microscopic structures. CUBIC has been successfully applied to a wide range of tissues, including murine pancreas (Tainaka *et al*. 2014, Susaki *et al*. 2015). Together, our data suggest that , within islets, reduction in insulin expression in beta cells precedes islet loss in cats with DM and that a CUBIC pipeline can be successfully adapted for use with surplus pancreatic tissue collected during the course of diagnostic postmortem examination (PME) of feline patients. This offers the possibility of utilising this powerful imaging technique in future studies of feline DM.

## MATERIALS AND METHODS

### Study population

Samples for the study were selected from the anatomic pathology archives of three institutions offering veterinary diagnostic pathology services. Cats with DM were defined as those where a diagnosis of DM was reached clinically before biopsies being taken and/or death (in the case of post mortem submissions), and where DM was therefore recorded as a diagnosis on the pathology submission form. Any diabetic cats that also had an additional diagnosis recorded detailing conditions that could lead to DM (e.g. hypersomatotropism, hyperadrenocorticism, steroid use) were excluded from the study. The inclusion criteria for both the diabetic and control cats are listed in Data S1. Samples were collected from both surgical biopsies and PME of cats that either died naturally or were euthanased. Slides and history from each case were reviewed by a single board‐certified pathologist in order to exclude pancreatic pathology other than DM. This project received ethical approval from the institution of the corresponding author.

### Processing of samples

Formalin‐fixed paraffin‐embedded pancreatic tissue was processed following routine protocols. Five μm thickness sections were cut and mounted either on glass slides for haematoxylin and eosin staining or on coated glass slides (TOMO®) suitable for IHC and IF. Slides for IHC and IF were deparaffinised, re‐hydrated and antigens were retrieved in one step using an antigen retrieval machine (PT Link – Dako Omnis) with a high pH (9) antigen retrieval solution (EnVision FLEX Target Retrieval Solution, High pH, Dako Omnis).

### Immunohistochemistry

After antigen retrieval was completed, tissue sections on slides were circled using a PAP pen (Vector Laboratories – H400). IHC was performed using ImmPRESS® Duet Double Staining Polymer Kit (HRP Anti‐Mouse IgG‐brown, AP Anti‐Rabbit IgG‐magenta; Vector Laboratories), following the instructions provided with the kit. Briefly, endogenous peroxidases were quenched, followed by blocking with 2.5% normal horse serum for 1 hour. Primary antibody was applied at the appropriate concentration for 1 hour, followed by secondary antibodies for 30 minutes. The reaction was developed using ImmPACT™ DAB EqV substrate. Counterstaining was achieved by applying Mayer's haematoxylin. Slides were then dehydrated by incubating in increasing concentrations of ethanol, followed by xylene. Slides were then coverslipped using D.P.X. neutral mounting medium (Sigma‐Aldrich). IHC slides were digitally scanned at ×40 using a NanoZoomer 2.0‐RS (Hamamatsu). Analysis was carried out digitally using the NDP.View2 viewing software (Hamamatsu). Details of specific antibodies are provided in Data S2.

### Immunofluorescence

Following antigen retrieval as above, a PAP pen was used to circle the sections of tissue and a small amount of 2.5% normal horse serum was applied for 1 hour. Primary antibodies were diluted to the required concentration and slides were incubated in a humidified chamber at 4°C overnight. Subsequently sections were treated with fluorescent secondary antibodies and incubated for an hour in the dark. Nuclei were labelled with 4′,6‐diamidino‐2‐phenylindole (DAPI) for 5 minutes and coverslips were applied using Vectashield® Vibrance™, antifade mounting medium (Vector Laboratories). Slides were assessed using a fluorescence confocal microscope (Leica SP8). Images were collected using a ×40/1.3 oil objective. Details of specific antibodies are provided in Data S2.

Image analysis to assess the area of each field occupied by synaptophysin‐labelled islets and the insulin signal within these islet‐regions was conducted using MATLAB R2020b and the Image Processing Toolbox (MathWorks). To estimate the insulin content of islets, the anti‐insulin fluorescence signal was integrated within masks of the synaptophysin‐delineated islet areas in each image.

### CUBIC tissue clearing and imaging

Pancreas used for CUBIC was collected from selected cats undergoing PME. The animals either died spontaneously or were euthanased for reasons unrelated to this study. Pancreata were collected within 12 hours from death and fixed whole in 10% formalin for 8 to 12 hours at room temperature. Subsequently, tissue was transferred to phosphate‐buffered saline (PBS) and stored at 4°C until use. Tissue from the body of the pancreas fixed for 12 hours in formalin was cut to 1 cm × 1 cm × 0.5 cm sections and was subsequently cleared using a published protocol (Susaki *et al*. 2015, Nojima *et al*. 2017) previously adapted for veterinary species (Nagy *et al*. 2021). Briefly, tissue pieces were incubated with reagent 1 (R1a) solution for 3 days at 37°C on a shaker. The solution was refreshed daily. The sections were then blocked in 10% goat serum overnight at 4°C on a shaker. Primary antibodies were diluted to the required concentration in blocking buffer and samples were incubated for 4 days at 4°C on a shaker. Following washing, secondary antibodies diluted in blocking buffer were applied for 2 days at 4°C. DAPI was applied for 2 hours at room temperature and then the samples were incubated in reagent 2 (R2) solution at 37°C for 2 days. Details of specific antibodies are provided in Data S1. Samples were imaged in R2, using Ibidi dishes using a confocal microscope (Leica SP8). Images were captured using ×10, ×20 and ×40 objectives. Images were further processed using Fiji (Schindelin *et al*. 2012).

### Statistical analysis

Data were log10 transformed and assessed for variance homogeneity and distribution normality (P>0.05) using the Bartlett and Shapiro–Wilk tests, respectively. In all instances, transformed data exhibited homogeneous variance and normal distributions enabling pairwise comparison using the two‐sided, Dunnett's *t*‐test with alpha set at 0.05. Where appropriate, mean values are stated ±sd.

## RESULTS

### Study population

A total of nine diabetic and 10 non‐diabetic cats were included in this study. The diabetic cats were domestic shorthair (DSH) (n=5), domestic longhair (DLH) (n=1) Maine coon (n=1), Tonkinese (n=1) and one cat of unknown breed, whereas non‐diabetic breeds were DSH (n=7), DLH (n=2) and British blue (n=1). The mean age for the diabetic group was 11 years old (median: 13 years, range: 6 to 17 years), while the mean age for non‐diabetic cats was 10 years old (median: 10 years, range: 8 to 14 years). In the diabetic group, there were seven male neutered and two female neutered cats. In the control group, there were two male neutered, seven female neutered and one male entire cats. Only eight of the nine diabetic cats were included in the IF analysis. Pancreata from three separate non‐diabetic cats with no pancreatic pathology were collected prospectively for analysis via CUBIC. Two of these cats were male neutered DSH (one 16 years old and other of unknown age) and one was a male entire Ragdoll (3 months old).

### Overall islet mass is reduced in diabetic cats

**FIG 1:**
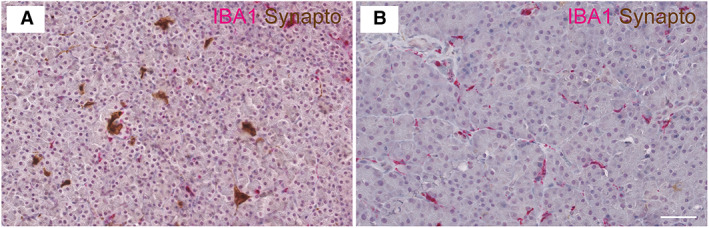
Overall islet mass is significantly lower in diabetic cats. IHC for synaptophysin (synapto; brown) and IBA1 (pink) in non‐diabetic (A) and diabetic (B) feline pancreas. Haematoxylin counterstain. Scale bar = 100 μm. Images are representative of 19 biological repeats. IHC, immunohistochemistry

**FIG 2:**
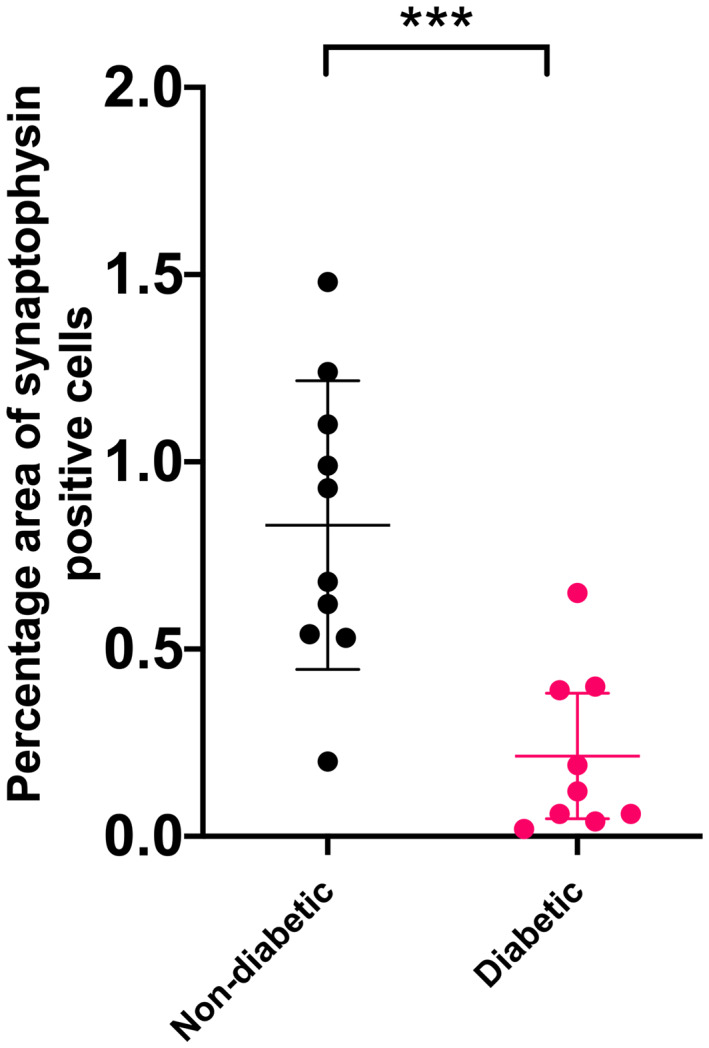
There are significantly fewer synaptophysin‐positive cells in diabetic compared with non‐diabetic cats. Scatter plot demonstrating significantly lower overall synaptophysin‐positive cells as a percentage of the pancreatic area examined in diabetic cats. Each dot represents the synaptophysin‐positive percentage area in an individual cat. Bars represent mean ±sd. ***P<0.001

Synaptophysin is an integral membrane glycoprotein found in presynaptic vesicles of neurons and neuroendocrine cells (Wiedenmann *et al*. 1986) that is used as a non‐specific marker for the different types of neuroendocrine cells comprising the pancreatic islets. As such, changes in the overall mass of synaptophysin‐positive cells can be interpreted as a surrogate read‐out of changes in islet mass. Examination of pancreatic sections stained immunohistochemically for synaptophysin revealed that diabetic cats have a significantly lower synaptophysin‐positive area compared with non‐diabetic cats (standard error=0.0389, *t* value=−4.490, P=0.0003) (Figs 1 and 2). Diabetic cats had a mean synaptophysin‐positive area of 0.3 ±0.2% of the whole pancreatic area examined, whereas non‐diabetic animals had a mean of 0.8 ±0.4%. Macrophages, identified by immunohistochemical staining for IBA1, were scattered throughout the endocrine and exocrine pancreas, and islet macrophage density was similar between control and diabetic cats (Fig 1).

### Insulin expression by β cells decreases before cell loss in diabetic cats

**FIG 3:**
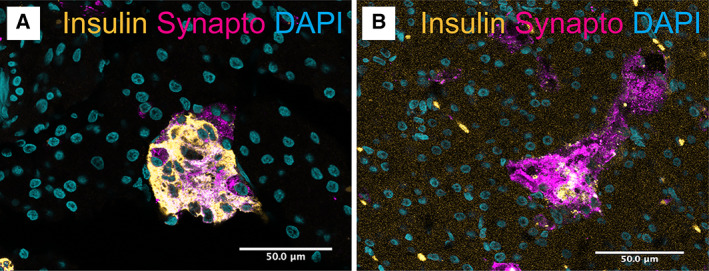
Insulin intensity is lower in diabetic cats. IF for insulin (gold), synaptophysin (synapto; magenta) and DNA (DAPI; cyan) in non‐diabetic (A) and diabetic (B) feline pancreas. Scale bar = 50 μm. Images are representative of 18 biological repeats. IF, immunofluorescence

**FIG 4:**
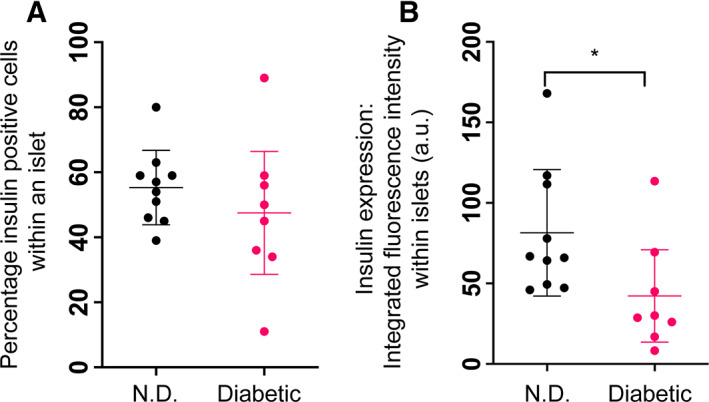
Whilst percentage of islet area comprising β cells is similar between non‐diabetic and diabetic cats, intensity of insulin expression is lower in diabetic cats. Scatter plots demonstrating that the islets of non‐diabetic (N.D.) and diabetic cats are composed of similar percentages of β cells (A), but this is accompanied by a significantly lower fluorescence intensity of the insulin signal within islet image regions in diabetic cats compared with non‐diabetic cats (B). Each dot represents an individual cat. Bars represent mean ±sd. *P<0.05

To assess the percentage of each islet comprising β cells, immunofluorescence staining for insulin was performed. The mean percentage of each islet that was composed of β cells, as inferred from immunofluorescence signal for insulin, was similar between non‐diabetic and diabetic cats. The mean islet area of non‐diabetic cats that was occupied by insulin positive cells was 55 ±11%, compared with 47 ±21% of the area in in diabetic cats (Figs 3 and 4). The distribution of percentage of insulin positive cells was much wider in the population of diabetic cats, consistent with the known heterogeneous nature of the disease (Fig 4A). Whilst the number of β cells, as indicated by insulin positive area, was similar between the two groups (standard error=8.1850, *t* value=−0.952, P=0.3548), the intensity of the insulin signal was significantly lower in diabetic cats compared with non‐diabetic individuals (standard error=0.1295, *t* value=−2.827, P=0.0121) (Fig 4B).

### CUBIC technology can be applied to optically clear and image feline pancreas

**FIG 5:**
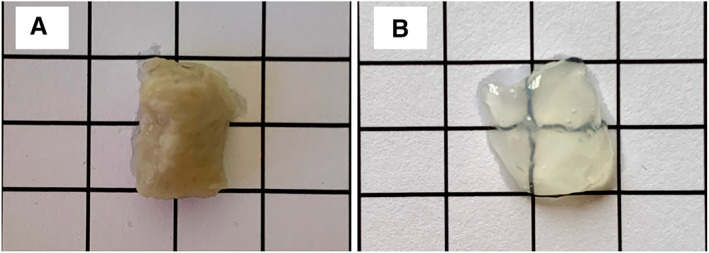
CUBIC provides effective optical clearance of formalin‐fixed feline pancreas. Formalin‐fixed non‐diabetic feline pancreas before (A) and after (B) CUBIC processing. Note that tissue piece has been inverted in second image. Images are representative of three biological repeats

CUBIC was used to optically clear formalin‐fixed tissue and was combined with IF for insulin to label pancreatic islets. CUBIC was successfully applied to non‐diabetic feline pancreas and a high level of macroscopic optical clearance was achieved (Fig 5). IF of CUBIC‐cleared feline pancreas allowed delineation of 3D microanatomy of healthy pancreatic islets by a combination of immunofluorescence staining for insulin together with DAPI staining for nuclei. This technique allowed assessment of islet 3D shape in an unprecedented level of detail: cutting away the tissue volume revealed insulin‐delineated cell groupings which could then be individually explored from all angles in 3D, offering the future opportunity for the islet and its constituent cells to be isolated and explored (Data S3).

## DISCUSSION

Using immunohistochemical staining for synaptophysin, this study demonstrates that diabetic cats have significantly lower synaptophysin‐positive pancreatic tissue areas compared with non‐diabetic cats. This finding corroborates that of previous investigators who documented that a population of diabetic cats showed an overall decrease in islet area, as determined by staining for insulin, compared with their non‐diabetic counterparts (Zini *et al*. 2016). This likely reflected a population of cats with DM at the final stage identified in human T2DM.

In DM, human pancreatic islets have been shown to go through several distinct stages before reaching the end‐point of islet destruction. Initially, as a response to insulin resistance, an expansion in β cell mass can be observed in the islets of non‐diabetic obese individuals (Rhodes 2005, Saisho *et al*. 2013). This stage is also known as prediabetes. The demand for insulin remains high, due to persistent hyperglycaemia coupled with insulin resistance, and a decline in β cell function and β cell numbers ensues. At this point, islets possess seemingly normal numbers of β cells, but they are unable to produce adequate amounts of insulin (Yagihashi *et al*. 2016). Ultimately, the number of β cells start to drastically decline (Butler *et al*. 2003, Rahier *et al*. 2008). These findings have been instrumental in explaining the pathogenesis of T2DM and provide a solid basis for the classification of the clinical course of human disease. By contrast, the changes affecting islets and β cells in feline DM are not as well‐characterised as they are in humans.

Using immunofluorescence and computational approaches, we show that the remaining islets of known diabetic cats exhibit a decrease in insulin signal intensity compared with control cats. We postulate that this reflects decreased β cell function, or β cell exhaustion, in spite of a similar proportion of the islet being occupied by insulin positive (β) cells. In such affected islets, this may reflect a feline equivalent to the stage in the pathogenesis of human DM that is temporally located between prediabetes and irreversible loss of islets. A prediabetic stage and consequent loss of functional islet mass have been identified in cats, and our data suggest that a decrease in insulin signal intensity is potentially likely one of the histopathological features of the prediabetic stage (Appleton *et al*. 2001, O'Brien 2002, Henson & O'Brien 2006, Reeve‐Johnson *et al*. 2016). This study, therefore, demonstrates the utility of a combined imaging‐ and computational‐ approach in facilitating identification of more subtle stages of functional and anatomical islet deterioration. This is particularly valuable at a point in disease progression when cause and consequence can be challenging to unravel, given that hyperglycaemia can also induce β cell dysfunction (Zini *et al*. 2009). Ultimately identification of such step‐wise loss in islet function will provide the basis for potential earlier testing and/or intervention in cats of predisposed breeds or exhibiting other risk factors such as obesity.

One notable limitation of this study is that cases were retrieved from pathology archives and consequently limited clinical data are available. In order to further characterise the prediabetic stage, histopathological findings, such as the ones presented in this study, should be correlated to clinical data such as weight, body condition score, blood glucose, blood glucose curves and fructosamine in a larger cohort of patients in which patients with concurrent diseases representing potential underlying causes of DM, such as hypersomatotropism, are identified and excluded from analysis. Serum insulin measurements would also be extremely valuable when trying to stage histopathological changes, as serum insulin has been demonstrated to be a reliable indicator of insulin resistance in cats (Appleton *et al*. 2005). Unfortunately, these data were unavailable for the majority of cases in our study. Given that feline diabetic remission is only possible if there is persistence of functional β cells, a further focus for future study should be to relate the findings presented here to study of the pathogenesis of diabetic remission.

Utilisation of CUBIC presents some significant technical challenges and limitations that necessitate revision of pre‐existing tissue collection procedures. As with routine histological approaches, pancreatic tissue used for CUBIC needs to be collected promptly after death or at biopsy, as it tends to autolyse quickly. Pancreatic tissue to be utilised for a CUBIC pipeline then requires brief formalin fixation limited to 10 to 12 hours before transfer to PBS, as under and overfixation impact negatively on the level of optical clearing achieved (authors' unpublished observation). Furthermore, some antibodies are not compatible with the CUBIC tissue clearing protocol and so storage of tissue for CUBIC tissue clearing must always be performed alongside collection of tissue for routine histopathological analyses.

Our study highlights CUBIC as a valuable tool to investigate the 3D islet microanatomy of cats. This opens up a wealth of possibilities to explore other aspects of the islet microenvironment in 3D. For example, in humans and rodents, clearing techniques have been applied to the pancreas to highlight several features of the microenvironment both in health and disease. Some of these studies have focused on differences in neuronal and vascular network in diabetic and healthy islets (Lee *et al*. 2014, Butterworth *et al*. 2018), while others focused on the endocrine cell composition of islets (Tang *et al*. 2018). Using a CUBIC pipeline, all these factors could be investigated in healthy cats, in order to provide information on blood supply, innervation and endocrine cell composition of non‐diabetic islet microenvironment. These aspects of canine and feline islets would provide useful benchmarks for future pathological studies.

The pancreatic islet microenvironment represents an extremely dynamic and complex biological equilibrium. Studying its intricacies highlights both normal islet physiology and pathological mechanisms underpinning DM. Here we highlight the benefits of a multimodal approach to study the feline pancreas in order to better understand disease pathogenesis. In particular, we identify pathological evidence to support the theory that, within individual islets, a decrease in β cell function likely precedes islet loss in diabetic cats, as it is the case in human T2DM, and we demonstrate the utility of a CUBIC tissue clearing pipeline to interrogate the histoanatomy of the feline pancreas in 3D in unprecedented detail.

### Conflict of interest

None of the authors of this article has a financial or personal relationship with other people or organisations that could inappropriately influence or bias the content of the paper.

## Author contributions

**Valeria Bergomi:** Conceptualization (supporting); data curation (lead); formal analysis (lead); investigation (lead); methodology (lead); project administration (supporting); software (supporting); validation (lead); visualization (lead); writing – original draft (lead); writing – review and editing (supporting). **Sam Beck:** Investigation (supporting); resources (supporting); writing – review and editing (supporting). **Melanie Dobromylskyj:** Investigation (supporting); resources (supporting); writing – review and editing (supporting). **Lucy Davison:** Conceptualization (lead); data curation (supporting); formal analysis (supporting); funding acquisition (lead); investigation (supporting); supervision (supporting); writing – review and editing (supporting). **John Wills:** Data curation (lead); formal analysis (lead); funding acquisition (supporting); investigation (supporting); methodology (lead); software (lead); supervision (lead); validation (lead); visualization (lead); writing – review and editing (supporting). **Katherine Hughes:** Conceptualization (lead); data curation (lead); formal analysis (lead); funding acquisition (lead); investigation (lead); methodology (lead); project administration (lead); resources (lead); supervision (lead); validation (lead); visualization (lead); writing – original draft (supporting); writing – review and editing (lead).

## Supporting information


**Data S1.** Table documenting inclusion criteria for diabetic and non‐diabetic cats.
**Data S2.** Table documenting antibodies used for immunohistochemistry, immunofluorescence and CUBIC

**Data S3.** Three‐dimensional rendering (gold) demonstrating the morphology of a feline pancreatic islet delineated by insulin (magenta) in CUBIC‐cleared feline pancreas delineated by nuclear stain DAPI (cyan)
