# Supplementary material for: Insulin expression in β cells is reduced within islets before islet loss in diabetic cats
Source: J Small Anim Pract. 2022 Aug 19;63(11):809–15. doi: 10.1111/jsap.13541 (PMC9804985; doi:10.1111/jsap.13541)
Supplement: Supplementary file 1 — Data S1. Table documenting inclusion criteria for diabetic and non‐diabetic cats. Data S2. Table documenting antibodies used for immunohistochemistry, immunofluorescence and CUBIC [file JSAP-63-809-s001.docx]

Supplementary data 1 Inclusion criteria for diabetic and non-diabetic cats.

| Criteria | Conditions for inclusion |
| --- | --- |
| Diagnosis of DM (for diabetic samples only) | DM diagnosed clinically prior to biopsy/PM |
| Death to collection interval | Maximum 36 hours |
| Storage of carcass | Chilled |
| Comorbidities | No pancreatic pathology (e.g. pancreatitis, neoplasia) recorded on clinical notes (where available) or present in the plane(s) of tissue examined histologically  If known, cats with conditions that could cause DM (e.g hypersomatotropism, prolonged steroid use, etc.) were excluded |
| Others | No signs of pancreatic autolysis |

Supplementary data 2 Antibodies for immunohistochemistry, immunofluorescence and CUBIC.

| Antibody | Brand and clone | Concentration IHC/IF | Concentration CUBIC |
| --- | --- | --- | --- |
| Monoclonal mouse anti-human synaptophysin | Agilent Dako  DAK-SYNAP | 1:2000 (IHC)  1:1200 (IF) | n/a |
| Insulin rabbit mAb | Cell Signalling Technology  C27C9 | 1:15000 (IF) | 1:800 |
| Goat anti-rabbit fluorescent IgG Alexa Fluor™ Plus 647 | ThermoFisher Scientific  AB_2633282 | n/a | 1:500 |
| Goat anti-mouse fluorescent IgG Alexa Fluor™ Plus 647 | ThermoFisher Scientific  AB_263277 | 1:500 | n/a |
| Goat anti-rabbit fluorescent IgG Alexa Fluor™ 568 | ThermoFisher Scientific  AB_143157 | 1:500 | n/a |
| 4’,6-diamidino-2-phenylindole (DAPI) | Sigma-Aldrich | 1:1000 (IF) | 1:1000 |
